# Contactless monitoring of heart and respiratory rate in anesthetized pigs using infrared thermography

**DOI:** 10.1371/journal.pone.0224747

**Published:** 2019-11-06

**Authors:** Carina Barbosa Pereira, Henriette Dohmeier, Janosch Kunczik, Nadine Hochhausen, René Tolba, Michael Czaplik

**Affiliations:** 1 Department of Anesthesiology, Faculty of Medicine, RWTH Aachen University, Aachen, NRW, Germany; 2 Institute for Laboratory Animal Science and Experimental Surgery, Faculty of Medicine, RWTH Aachen University, Aachen, NRW, Germany; Central South University, The Third Xiang Ya Hospital, CHINA

## Abstract

Pig experiments have played an important role in medical breakthroughs during the last century. In fact, pigs are one of the major animal species used in translational research, surgical models and procedural training due to their anatomical and physiological similarities to humans. To ensure high bioethical standards in animal trials, new directives have been implemented, among others, to refine the procedures and minimize animals’ stress and pain. This paper presents a contactless motion-based approach for monitoring cardiorespiratory signals (heart rate and respiratory rate) in anesthetized pigs using infrared thermography. Heart rate monitoring is estimated by measuring the vibrations (precordial motion) of the chest caused by the heartbeat. Respiratory rate, in turn, is computed by measuring the mechanical chest movements that accompany the respiratory cycle. To test the feasibility of this approach, thermal videos of 17 anesthetized pigs were acquired and analyzed. A high agreement between infrared thermography and a gold standard (electrocardiography and capnography-derived respiratory rate) was achieved. The mean absolute error averaged 3.43 ± 3.05 bpm and 0.27 ± 0.48 breaths/min for heart rate and respiratory rate, respectively. In sum, infrared thermography is capable of assessing cardiorespiratory signals in pigs. Future work should be conducted to evaluate infared thermography capability of capturing information for long term monitoring of research animals in a diverse set of facilities.

## Introduction

Animal research has revealed a lot of medical insights and fundamental discoveries as humans search for evidence of the cause and treatment of diseases [1].

To provide the best animal welfare, measurable indicators of stress, such as heart rate (HR) and respiratory rate (RR), body temperature or behavioral indicators should be recorded in animal trials. In chronic animal studies, vital parameter assessment usually requires implantation of a telemetry sensor. But the initial surgical procedure itself causes additional stress, discomfort and limited mobility and therefore leads to reduced animal welfare. In short-term experiments with animals undergoing anesthesia, the monitoring of HR and RR is performed using electrocardiography (ECG) or photoplethysmography (PPG). Other parameters such as emotional state, pain or locomotion capability are currently assessed subjectively [2]. Nevertheless, ethical and legal obligations include adequate monitoring of animal health and reduction of suffering whenever possible.

Animal welfare is an important social issue in most European countries that build the consensus to restrict animal experiments to the necessary minimum [3]. The objective is to reduce the number of animals used in research through statistical and methodological improvements, to replace animal experiments with alternative methods whenever possible and to refine trials by minimizing the harmful effects on laboratory animals [4]. Alternatives that completely replace the use of animals, such as tissue cultures, microorganisms or computer models still do not make animal research unnecessary. Therefore, the focus is on the further reduction and refinement of animal experiments [5]. As long as complete replacement remains out of reach, we, as responsible researchers, have to work on refinement and reduction. A wide variety of technologies have been investigated to achieve refinement in animal experiments. Thermal imaging, also known as infrared thermography (IRT), is believed to be an auspicious technique for evaluating HR and RR [6–9] hemodynamics [10] and thermoregulation [11]. IRT is a non-harming method that does not even need light to detect the radiation that the body emits by its very nature [10]. With this background and to satisfy the high demand for non-debilitating and contactless monitoring modalities, we investigated the application of IRT in animal experiments with anesthetized pigs to compute their HR and RR.

## Materials and methods

**Fig 1:**
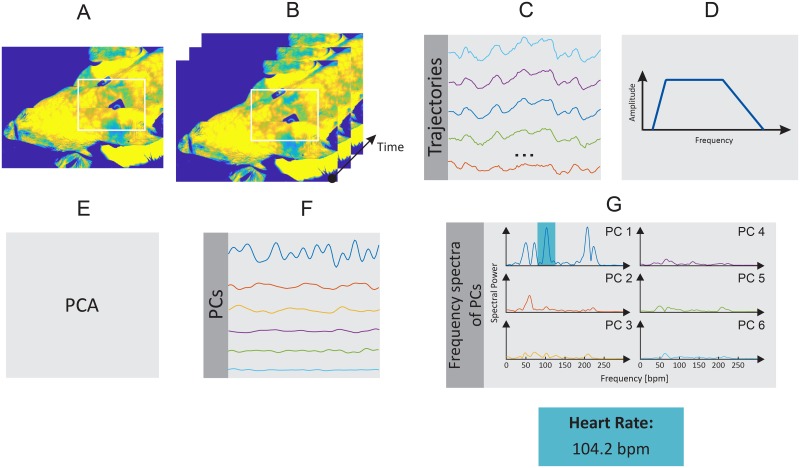
Schematic overview of the HR algorithm. A: Selection of regions of interest (ROI). B: Detection and tracking of feature points. C: Extraction of feature points’ trajectories. D: Temporal filtering. E: Blind source separation via principal component analysis (PCA). F: Rank principal components (PCs) based on their variance. G: Computation of frequency spectra and estimation of HR.

**Fig 2:**
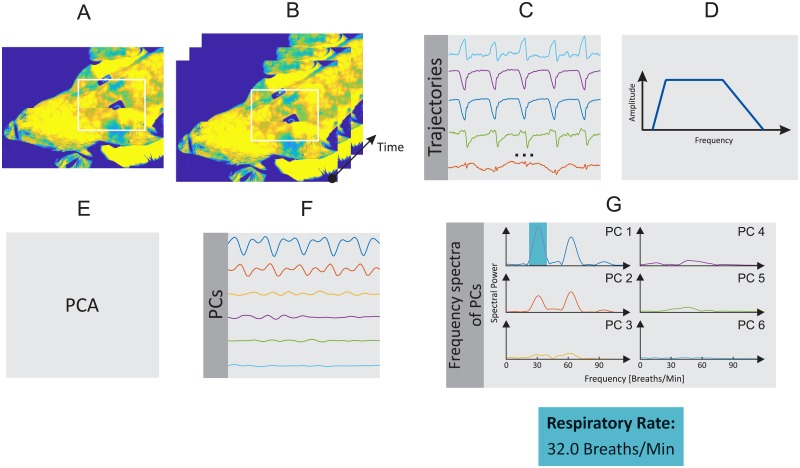
Schematic overview of the RR algorithm. A: Selection of ROI. B: Detection and tracking of feature points. C: Extraction of feature points’ trajectories. D: Temporal filtering. E: Blind source separation via PCA. F: Rank PCs based on their variance. G: Computation of frequency spectra and estimation of RR.

In the present study, the HR and RR of the piglets were estimated using a motion-based computer vision method. HR assessment is based on the weaker vibrations (precordial motion) of the chest caused by the heartbeat. RR, in turn, is estimated by measuring the mechanical chest movements that accompany the respiratory cycle. Figs 1 and 2 show a schematic overview of the motion-based algorithm, which is composed of six main steps, which are described in detail below. This approach was implemented in MATLAB (MATLAB 2018a, the MathWorks Inc., Natick, MA, USA), and data were analyzed offline.

### Image preprocessing

In the first step, the animal was segmented from the background using the multilevel Otsu’s algorithm, which estimates optimal threshold values using discriminant analysis. In short, the algorithm maximizes between-class separability while minimizing within-class variability [12]. Additionally, the contrast of the thermograms was enhanced by linearly stretching the temperature levels to a new range. Image preprocessing was performed for each single frame of the thermal video.

### Selection of region of interest

The second step of the algorithm consists in selecting the region of interest (ROI), that is, the chest of the pig, in the first frame of the thermal video. Based on empirical knowledge, a ROI with approximately 27500 pixels (about 3.5% of the whole thermogram, considering a resolution of 1024×768 pixels) is enough for signal extraction.

### Motion tracking

To track the chest surface motion owing to the beating heart and respiration, *N* feature points were identified within the ROI in the first frame of the video and, afterward, tracked over time. Feature points are usually found in corners (intensity variation is strong along all directions) and edges (intensity variation is strong in one direction). In this paper, the Shi-Tomasi corner detector [13] was applied. The maximum number of feature points used for tracking can be defined by the user. In this work, the candidate features were ranked according to their strength, and only the *N* = 100 strongest features were selected.

The position of the feature points was given as an input to the Kanade-Lucas-Tomasi tracker. This tracked their trajectories over time using optical flow. To preserve only relevant trajectories, the signal quality of the trajectories was analyzed. Note that some feature points might present a poor texture and, as a result, lead to erratic trajectories. Therefore, those points whose trajectories between successive frames transcended a certain percentile were neglected. This selection allowed the preservation of only the most stable feature points in the following steps. In addition, motion trajectories were divided into their subcomponents: vertical and horizontal. The component containing the main chest movement owing to heartbeat and respiration was selected for further analysis.

### Temporal filtering

Not all trajectory frequencies are necessary for RR and HR estimation. Therefore, the signal frequencies were constrained to the expected ranges to remove noise. The vital parameters of pigs vary according to their age. Under normal conditions, the RR of weaned pigs ranges from 25 to 40 breaths/min, and their HR ranges from 90 to 100 beats per minute (bpm). Since the induced acute lung injury leads to increased HRs, a 50th-order band-pass finite impulse response (FIR) filter with a passband of [1.15 5.6] Hz was used. For the RR, a 50th-order FIR band-pass filter with a lower and upper 3 dB cutoff frequency of 0.25 Hz and 2 Hz was applied. Both filters were designed in MATLAB using the Parks-McClellan algorithm: in both the density factor was set to 20. Note that the upper 3 dB cutoff frequency was defined to include the RR/HR first harmonic since it provides significant information for an accurate peak detection.

### Principal component analysis decomposition

Heartbeat and respiration, which cause chest movement, are the primary signals of interest. However, the trajectories of the feature points also include other components that were not filtered in the previous step. Thus, to separate a set of main dimensions along where the position of the chest varies, blind source separation based on principal component analysis (PCA) was used. PCA reduces an initial large set of variables into a smaller set of linearly uncorrelated variables called principal components. These were sorted in a descending order of variation. Thus, the first principal component contained most of the variations presented in the original dataset. In this study, only the first six components were computed. In addition, PCA was done by eigenvalue decomposition of the covariance matrix. During video acquisition, some movements or tracking errors can influence the position of the vectors, by adding variance, and consequently the PCA decomposition. To overcome this issue, a determined percentage of the feature point trajectories with the largest L2-norms were not included in the PCA.

### Principal component selection

The last step consisted in selecting the component used to extract the respiratory/pulse frequency. For this purpose, signal periodicity, based on the frequency spectra, was computed. However, before the fast Fourier transform (FFT) was applied, the six principal components were Hamming windowed and zero padded. As well known, a rectangular window applied to a signal in the time domain might cause distortions in the frequency domain. These can be minimized with a smoother window shape (Hamming window) since it de-emphasizes the edges and reduces their effects. To calculate the periodicity of the principal components, the peak-to-total ratio approach was used; it corresponds to the ratio of the frequencies within a range of 0.05 Hz around the dominant frequency and its first harmonic in the frequency spectrum to the total spectral density. While a high periodicity denotes a signal with a clear dominant frequency, a low periodicity indicates a signal containing mostly aperiodic noise. Directly after the principal component was selected, which has the highest periodicity, the clearest main frequency was chosen as RR/HR. Finally, to avoid outliers in the RR/HR signal, a median filter was used.

## Experimental protocol

The data used for this work have been assessed as part of a lager study to comply with the three Rs principle (replacement, refinement and reduction). The study complies with the experimental protocol that was approved by the governmental animal care and use institution “*Landesamt für Natur, Umwelt und Verbraucherschutz NRW*” (Germany, AZ84-02.04.2013.A200). All procedures were performed according to the German Animal Welfare Law. All animals in the present study received human care according to the principles of the “Guide for the Care and Use of Laboratory Animals” (8^*th*^ edition, NIH Publication, 2011, USA).

For the additional measurements, 17 of the study pigs (weighing 37.4 ± 3.22 kg) were randomly selected. A veterinarian confirmed the absence of preexisting diseases. For the main investigation, the unshaved pigs were put under anesthesia, intubated and placed in a supine position. To maintain the narcosis, a total intravenous anesthesia with thiopental (7-12 mg kg^−1^ h^−1^) and fentanyl (6-10 *μ*g kg^−1^ h^−1^) was continuously delivered. Additionally, the fluid loss was compensated with Ringer’s solution (5 ml kg^−1^ h^−1^) and adjusted to the animals’ needs. For volume balance, a transurethral catheter was placed. ECG was derived simultaneously as a gold standard (GS) for HR calculation. For this purpose, a patient monitor system (AS/3, Datex Ohmeda, Helsinki, Finland) was utilized. The GS of the RR was delivered by capnography and obtained by the anesthesia machine (Cato, Dräger Medical, Lübeck, Germany). The animals were ventilated mechanically in a volume-controlled mode with a tidal volume of 8 ml kg^−1^ body weight, an inspiration-expiration ratio of 1:1 and an initial positive end-expiratory pressure (PEEP) of 5 mbar. After the acute experiment was finished, the animals were euthanized by an overdose of Pentobarbital.

In this animal trial, IRT sequences were recorded for a duration of 2 minutes by using a long-wave infrared (LWIR) camera, VarioCAM^®^ HD head 820S/30 (InfraTec GmbH, Dresden, Germany), pre-calibrated, with a thermal sensitivity of 0.03°C at 30°C. The camera detects wavelengths at a spectral range of 7.5 to 14 *μ*m and presents a spatial resolution of 1024×768 pixels. The described device allows a capturing rate of 30 frames per second (fps), thus each video sequence was composed of 3600 frames. For image collection, the camera was positioned at the head’s side of the animal and connected to a standard laptop computer. The distance between the camera and the animal was about 1.5 m. In addition, the parameter emissivity was set to 0.98. According to a study from Soerensen *et al*. [14], it is valid to use the human skin emissivity (0.98) for measurements on pigs. Regarding ambient operating room temperature, it was approximately 23°C.

## Results

### Estimation of heart rate

Table 1 shows the performance of the algorithm developed for the estimation of the HR in IRT sequences of anesthetized pigs. The HR of the pigs measured as a GS averaged 131.29 ± 22.95 bpm. The HR measured with the IRT averaged 131.43 ± 21.74 bpm. The comparison of both monitoring techniques of the mean absolute error amounted to 3.43 bpm, and therefore, the mean relative error was 2.69%.

**Table 1:** Results for HR estimation in infrared thermograms from pigs.

Heart Rate				
ID	IRT [bpm]	GS [bpm]	Abs. Error [bpm]	Rel. Error
1	142.60	138	4.60	3.33%
2	137.70	142	4.30	3.03%
3	107.90	108	0.10	0.09%
4	156.90	158	1.10	0.70%
5	154.10	154	0.10	0.06%
6	157.95	158	0.05	0.03%
7	104.00	103	1.00	0.97%
8	115.63	113	2.63	2.33%
9	164.03	167	2.97	1.78%
10	104.07	101	3.07	3.04%
11	94.56	92	2.56	2.78%
12	120.00	129	9.00	6.98%
13	116.66	120	3.34	2.78%
14	149.46	154	4.54	2.95%
15	142.43	145	2.57	1.77%
16	135.47	124	11.47	9.25%
17	130.86	126	4.86	3.86%
Mean	131.43	131.29	3.43	2.69%
SD	21.75	22.96	3.05	2.41%

Abs. Error: Absolute Error; Rel. Error: Relative Error

**Fig 3:**
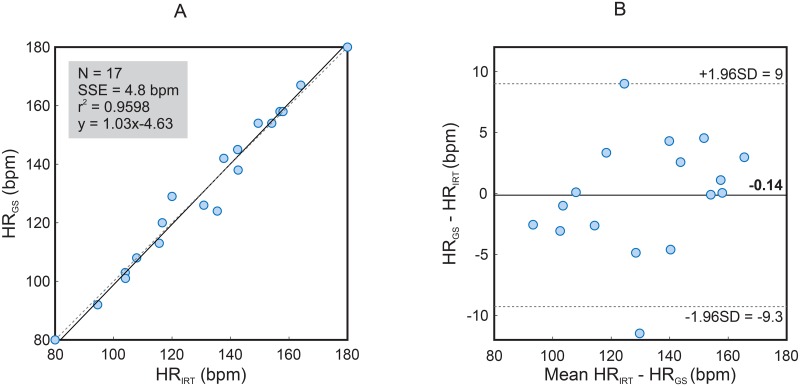
Correlation plot and Bland-Altman plot comparing HR assessed with infrared thermography (HR_*IRT*_) and HR assessed using ECG (HR_*GS*_). The plots include the data from all the 17 animals. A: The plot on the left shows an R-squared of 0.9598 and a sum of squared errors of 4.8 bpm. B: The graph on the right presents a bias of -0.14 bpm (solid line), and the 95% limits of agreement vary between -9.3 and 9.0 bpm (dashed lines).

Fig 3 presents a correlation plot and a Bland-Altman plot comparing both monitoring techniques, the IRT and the GS assessed using an ECG, in terms of HR. They comprise the data from all the 17 pigs. As shown by the results, the R-squared (coefficient of determination) accounted 0.96, and the sum of squared errors (SSE) averaged 0.48 bpm. The Bland-Altman plot registered a mean difference of -0.14 bpm. The 95% limits of agreement reached from -9 to 9 bpm. S1 Video is an illustrative example of the performance of the algorithm. It corresponds to animal number 4.

### Estimation of respiratory rate

Table 2 presents the results of the estimation of the RR with the corresponding algorithm. On average, the RR of the anesthetized animals given by the ventilator as GS amounted to 31.41 ± 3.10 breaths/min. The IRT-ascertained RR measured 31.29 ± 3.06 breaths/min. Comparing both monitoring techniques, the mean absolute error accounted 0.27 breaths/min and the mean relative error 0.79%.

**Table 2:** Results for RR estimation in infrared thermograms from pigs.

Respiratory Rate				
ID	IRT [breaths/min]	GS [breaths/min]	Abs. Error [breaths/min]	Rel. Error
1	32.14	32	0.14	0.44%
2	32.12	32	0.12	0.38%
3	31.66	32	0.34	1.07%
4	31.64	32	0.36	1.13%
5	33.00	35	2.00	5.71%
6	33.93	34	0.07	0.20%
7	24.96	25	0.04	0.18%
8	29.81	30	0.19	0.64%
9	36.69	36	0.69	1.92%
10	32.03	32	0.03	0.08%
11	33.88	34	0.12	0.35%
12	30.16	30	0.16	0.54%
13	30.08	30	0.08	0.27%
14	29.87	30	0.13	0.43%
15	25.03	25	0.03	0.12%
16	29.99	30	0.01	0.03%
17	35.01	35	0.01	0.03%
Mean	31.29	31.41	0.27	0.79%
SD	3.06	3.10	0.48	1.36%

Abs. Error: Absolute Error; Rel. Error: Relative Error

**Fig 4:**
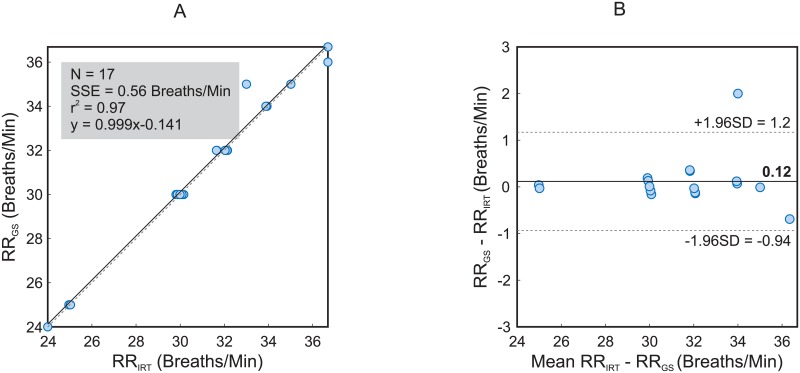
Correlation plot and Bland-Altman plot comparing RR assessed with infrared thermography (RR_*IRT*_) and RR assessed using the ventilator (RR_*GS*_). The plots include the data from all the 17 animals. A: The plot on the left shows an R-squared of 0.97 and a sum of squared errors of 0.56 breaths/min. B: The graph on the right presents a bias of -0.14 breaths/min (solid line), and the 95% limits of agreement vary between -0.9 and 1.2 breaths/min (dashed lines).

Fig 4 represents the correlation plot and the Bland-Altman plot comparing the two monitoring techniques concerning RR. As in Fig 3, the data from all the 17 animals were analyzed. The R-squared amounted to 0.97 and the SSE 0.56 breaths/min. The Bland-Altman plot displayed a mean difference of -0.12 breaths/min. The 95% limits of agreement ranged from -0.9 to 1.2 breaths/min. S2 Video is an illustrative example of the performance of the algorithm. It corresponds to pig number 4.

## Discussion

This work illustrates the capacity of IRT to precisely measure HR and RR in anesthetized pigs. It is an extension of [6], where the capability of IRT to assess RR was demonstrated. On the one hand, both parameters are important indicators for detecting stress in laboratory animals; on the other hand, the possibility of contactless detection of these parameters through IRT offers an auspicious method to refine animal experiments (three Rs principle). To test the method and the algorithm, IRT sequences were recorded during an animal trial involving anesthetized pigs.

HR and RR are important parameters for evaluating the health condition and well-being not only of humans but also of laboratory animals. An abnormal RR, sound variation or atypical waveforms, like depths or rhythm variations, are strong indicators of severe complications. Detection of abnormalities of the HR and cardiac rhythm not only serve as a stress indicator but also are valuable diagnostic instruments for cardiac diseases. An increase in RR and HR might be associated with pain, fear, anxiety and panic. Especially in chronic animal experiments, passive, continuous monitoring such as IRT is highly demanded.

In this paper, we show an approach to extract the RR from chest movements and the HR from chest vibrations. GS- and IRT-derived HR and RR measures significantly corresponded with clinically negligible deviations. For the HR, the mean absolute error amounts to 3.43 bpm, the mean relative error 2.69%. The measurements of the RR even resulted in a higher accuracy, with a mean absolute error of 0.27 breaths/min and 0.79% for the mean relative error. Fig 3 displays the correlation and the Bland-Altman plot of both measurement techniques for the HR. The coefficient of determination accounts 0.96 and the sum of squared errors 4.8 bpm. The Bland-Altman plot confirms a good concordance between both techniques with 95% limits of agreement ranging from -9.3 to 9.0 bpm. Fig 4, just like Fig 3, shows the correlation and the Bland-Altman plot but for the assessment of the RR. The coefficient of determination measures 0.97 and the sum of squared errors 0.56 breaths/min. In addition, the Bland-Altman plot confirms a stronger coherence between both techniques with 95% limits of agreement ranging from -0.9 to 1.2 breaths/min.

Whereas the assessment of HR_*GS*_ and RR_*GS*_ was only conducted at one defined time point, several measures were obtained from IRT sequence analysis (the sampling rate of our algorithm is one value per second). Regarding RR, a strong agreement was obtained, as the animals were mechanically ventilated with a constant frequency ($\bar{RR_{GS}}=RR_{GS}\left(t\right)$ with 0 < *t* ≤ 2 min). By contrast, HR varied along the 2 min (spontaneous cardiovascular variability). Therefore, a single GS value (obtained at the beginning of the 2-min measurement) was compared to the mean HR_*IRT*_ (set of values denoting the variation in the beat-to-beat interval). This factor could have contributed to higher errors (2.69%) as well as higher limits of agreement in the Bland Altman plot (-9.3 to 9.0 bpm).

A lot of research has been conducted during the last years to refine animal experiments. Alternatives for a contactless assessment of the RR exist but either need a complex experimental setup (observation of thorax, measuring pressure differences), are sensitive to errors (analyze breathing sound [15]) or still irritate the animal (changes in air temperature in the nostrils [16, 17]). In 2018, Mutlu et al. [9] developed a technique for respiration monitoring in rodents using IRT. This work group used the fact, that the air temperature differs during a breathing cycle. During inhalation, cold air enters the nostrils, while warm air from the lung is exhaled. As GS, an intranasal pressure sensor was used. Altogether, the ascertained results were comparable. While this is a good alternative to measuring the RR, there is a disadvantage referring to the measurement technique and the aim to refine animal experiments. For data assessment, the nostrils always have to be in the recording field of the camera. This indicates an inevitable previous surgical procedure to restrain the rodent’s head by implanting a head plate to fixate it. In our investigation, the camera position was flexible. Due to the complex experimental setup for the main animal trial, a constant camera angle and position was not always possible. The placement of the optical setting was affected by the medical needs. First, the equipment could not prevent the anesthesiologist to perform its countless tasks according to the study protocol (e.g. administration of medicaments, blood analysis, among others). Secondly, the animal trial required several medical devices (e.g. ventilator, patient monitoring system, etc.) and equipment (e.g. tables to place the sterile objects), which led to a reduction of the space available. These factors prevent us to place the camera in a constant position, and to use the same angle. However, these challenging conditions are in fact positive, as they demonstrate the generalizability of our approach, which does not depend neither on the position of the camera nor on its angle.

In 2013, Zhao et al. [18] conducted remote measurements of both vital parameters in different living beings. In contrast to our study, they used a near-infrared technique, which always requires a light source. Our algorithm is based on motions and vibrations associated with respiration and heartbeat, whereas Zhao’s technique is a color-based method using blood perfusion. In addition, they presented the application in only one pig without a GS. Another promising alternative to contactless monitoring is remote PPG. It has a good accuracy of 90%, as Blanik et al. published in 2014 [19], but this technique also needs a light source for application. Taking stress reduction during animal trials into consideration, the method independency of an additional visible light source is an important factor and a big advantage of IRT. Within the context of contactless monitoring, radar technology also is present. Although this technology is able to detect HR and RR without any contact, it is highly sensitive to errors when it comes to the motion of the subject [20], making this a limiting factor. As reported, many studies about contactless detection of HR and RR have been conducted. To the best of our knowledge, this is the first study that measures the HR in pigs with IRT.

In this paper we focused on sedated, and thus, immobilized animals. However, assessment of vital parameters are also fundamental during chronic experiments using pigs. Within this context, it would be interesting to examine in future studies the transferability of this algorithm. Certainly, this is a challenging task. First, the chest is less or not visible. Secondly, a detection algorithm for an automatic identification of the ROI must be implemented. Thirdly, a robust algorithm must be integrated to track the animal (either for single or group-housing). Eventually, it shall be combined with the information of subcutaneous RFID (radio-frequency identification) tags. Fourthly, in this new scenario movement must be considered: thus, new monitoring strategies should be established (e.g.: the vitals can be only assessed when the pig is still or lying). Fifthly, during exploratory behavior pigs intensively use their respiratory organ altering their normal respiratory frequency.

IRT is still an expensive method, especially compared with visible imaging systems. Certainly, a major advantage of IRT is its independence from visible light. Therefore, vital parameters can me assessed without interrupting the day/night cycle. Another advantage of IRT is the detection of body temperature. The rise of body temperature indicates stress as well [21]. Heart rate variability (HRV) is also a stress parameter that is often investigated. In combination with remote PPG, it seems imaginable to evaluate HRV with IRT.

Besides, the increasing number of IRT applications in the last years should be considered, mostly in consumer-orientated devices, such as driver’s vision enhancement or home security. This development has led to a higher production rate and consequently a decrease in production costs. Like other technologies before, IRT cameras will follow the hype cycle and become more affordable and even better.

## Supporting information

S1 Video HR estimation. Illustrative example showing the performance of the HR estimation algorithm.
(MP4)

S2 Video RR estimation. Illustrative example showing the performance of the RR estimation algorithm.
(MP4)
